# Comparative profiling of extracellular vesicles and miRNA cargo from in vivo- and in vitro-derived bovine embryos during blastulation and hatching

**DOI:** 10.1186/s40104-026-01378-y

**Published:** 2026-04-13

**Authors:** Miguel A. Gutiérrez-Reinoso, Ioanna Martinez-Hormaza, Yat S. Wong, Constanza Aguilera, Joel Cabezas, Felipe Navarrete, Barbara Melo-Báez, Fidel O. Castro, Lleretny Rodriguez-Alvarez

**Affiliations:** 1 Departamento de Ciencia Animal, Facultad de Ciencias Veterinarias, Universidad de Concepción, Chillán, Chile; 2 Facultad de Ciencias Agropecuarias y Recursos Naturales, Medicina Veterinaria, Universidad Técnica de Cotopaxi, Latacunga, Ecuador; 3 Facultad de Medicina Veterinaria y Agronomía, Universidad de Las Américas, Providencia, Chile; 4 Facultad de Medicina Veterinaria, Universidad San Sebastián, Concepción, Chile

**Keywords:** Blastulation, Bovine embryo, Embryo–maternal communication, Extracellular vesicles, Hatching, In vitro fertilization, MicroRNA

## Abstract

**Background:**

Extracellular vesicles (EVs) represent an important component of embryo–maternal communication by conveying molecular signals that reflect the embryo’s physiological state and developmental competence. However, the combined impact of embryonic origin and developmental stage on EV molecular composition has remained largely unexplored. In this study, a comparison is presented for the first time between EVs secreted by bovine embryos produced in vivo (IVV) and in vitro (IVP) during the blastulation and hatching stages, providing evidence of how these factors shape their biological profiles.

**Results:**

IVV embryos exhibited higher developmental competence and secreted larger EVs whose concentrations remained stable across developmental windows. In contrast, IVP embryos released smaller and more abundant vesicles, particularly during hatching, indicating origin- and stage-specific regulation of EV output. Distinct miRNA profiles clearly separated both embryo types. EVs from IVV embryos were enriched in miRNAs associated with implantation and lineage specification (e.g., miR-124, miR-125, miR-181), whereas EVs from IVP embryos contained higher levels of miRNAs linked to stress response, apoptosis, and the unfolded protein response (e.g., miR-23b, miR-92a, miR-409). Consistently, functional enrichment analyses revealed that IVV-derived miRNAs targeted pathways related to immune modulation and purinergic signaling, while IVP-derived miRNAs were associated with calcium transport and endoplasmic reticulum stress pathways. Together, these differences point to divergent regulatory programs shaped simultaneously by embryonic origin and developmental progression.

**Conclusions:**

Embryonic origin and developmental stage influence the biophysical properties and miRNA composition of embryo-secreted EVs, reflecting distinct developmental trajectories between IVV and IVP embryos. This study provides the first direct evidence that embryonic origin significantly modulates EV physicochemical features and miRNA cargo, highlighting their role in embryo–maternal communication and supporting the use of EV-derived miRNAs as novel non-invasive biomarkers of embryo quality and developmental competence.

**Supplementary Information:**

The online version contains supplementary material available at 10.1186/s40104-026-01378-y.

## Introduction

Assisted reproductive technologies (ART) are crucial to the bovine industry because they accelerate the propagation of genetically superior livestock. Recently, a shift has occurred from in vivo-produced (IVV) to in vitro-produced (IVP) embryos as the preferred strategy for maximizing genetic gains in cattle [1]. Despite notable advances in IVP protocols and the optimization of culture media to enhance gamete and embryo competence, the cryotolerance and post-transfer pregnancy rates of IVP embryos remain lower than those of IVV embryos [2, 3].

The in vitro environment exerts a profound influence on early embryonic development, rendering them less competent than their in vivo counterparts. Morphological selection of blastocysts often fails to predict implantation potential, despite its widespread adoption for its simplicity and non-invasiveness. Even blastocysts considered of the highest quality (Grade 1) according to the International Embryo Technology Society's (IETS) grading [4] may exhibit compromised implantation capacity [5]. Most pregnancy losses occur during the peri-implantation period [6–8]. The in vitro environment impacts epigenetic and gene expression patterns, effects that may not be evident at the blastocyst stage but manifest later during embryo-maternal recognition processes [9–11], due to the compromised signaling required for successful maternal recognition [12, 13].

In bovines, interferon tau (IFN-τ) secreted by the embryo is the primary signal for maternal recognition of pregnancy. Despite the critical role of IFN-τ in pregnancy recognition, there is evidence that earlier signals secreted by the embryo may also prepare the maternal side for implantation [14, 15]. Pre-implantation embryos release extracellular vesicles (EVs), which are internalized by endometrial cells and participate in early embryo-maternal communication [16, 17].

EVs are nanometer-sized particles surrounded by a lipid bilayer and are naturally secreted into the extracellular environment by various cell types, including embryonic cells [18, 19]. During their biogenesis, EVs are loaded with diverse molecules, including proteins, lipids, RNAs, and DNA. They were originally described as mechanisms for cellular waste clearance [20]. However, EVs are now recognized as key mediators of intercellular communication, mediating the transfer of bioactive molecules to recipient cells through their molecular cargo [21, 22].

EVs have been linked to several physiological processes, including gamete and embryo development. Pre-implantation embryos from various species, including humans, pigs, mice, and cattle, secrete EVs [23–25]. The characteristics of EVs (size, concentration, and molecular cargo) vary according to the origin, developmental stage, and quality of the embryo [26]. The differential molecular composition of embryonic EVs reflects the embryo's developmental competence and may modulate embryo-maternal communication [27, 28].

Although the number of available studies remains limited, emerging evidence indicates that EV-mediated embryo–maternal communication differs between embryos produced in vitro and in vivo [17, 28–30]. It remains unclear how embryonic origin (IVV vs. IVP) and developmental stage (blastulation vs. hatching) together influence EV biogenesis, morphology, and molecular cargo. This lack of information limits our understanding of embryo–maternal communication and the molecular determinants of embryonic competence.

Under the hypothesis that both embryonic origin and developmental stage significantly modulate the population characteristics and miRNA content of EVs, this study aimed to characterize EV populations derived from IVV and IVP bovine embryos at distinct developmental stages. Through the direct comparison of EVs secreted by embryos produced IVV and IVP during the blastulation and hatching stages, this work constitutes the first comparative analysis integrating both variables. This approach provides new evidence on the mechanisms associated with the reduced competence of IVP embryos and establishes a biological framework for the development of non-invasive tools to assess embryonic competence and optimize embryo selection strategies.

## Materials and methods

All experiments were conducted at the University of Concepción, Campus Chillán, except for transmission electron microscopy, which was carried out at the Advanced Microscopy Facility of the Pontificia Universidad Católica de Chile.

### Experimental design

**Fig. 1:**
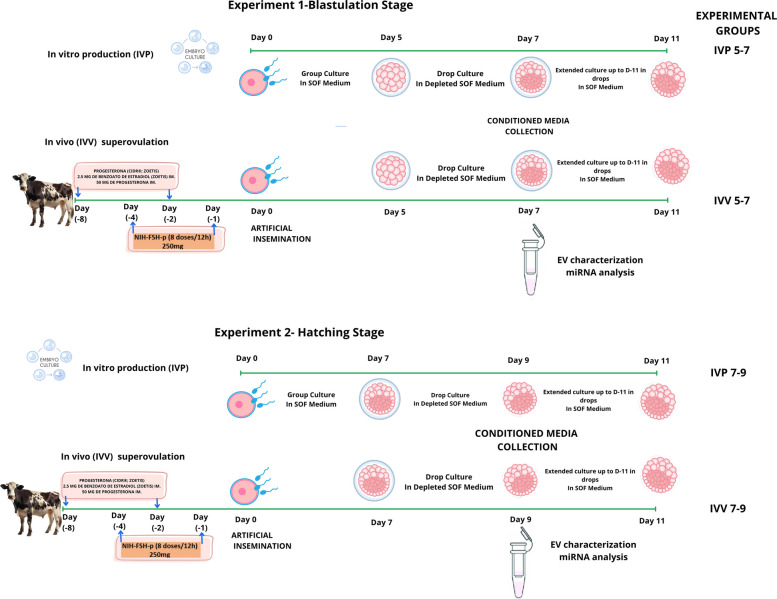
Experimental design for the evaluation of extracellular vesicle (EV) characteristics according to embryo origin (in vivo/in vitro) and developmental stage (blastulation/hatching)

Two experiments were conducted to characterize EV populations during specific windows of early embryonic development to elucidate how the characteristics of EVs secreted by bovine embryos are influenced by their origin (IVV or IVP) and developmental stage (blastulation and hatching) (Fig. 1). For both experiments, embryos were produced through IVV and IVP procedures. In Exp. 1, corresponding to the blastulation stage (d 5–7), a total of 30 IVV and 60 IVP embryos were obtained. In Exp. 2, which focused on the hatching stage (d 7–9), 30 IVV and 30 IVP embryos were produced.

#### Experiment 1

Evaluation of characteristics and miRNA profiles of EVs secreted during blastulation in IVP and IVV bovine embryos.

- IVV 5–7 Group: Morulae obtained via superovulation and artificial insemination.
- IVP 5–7 Group: Morulae generated through in vitro fertilization.

#### Experiment 2

Evaluation of characteristics and miRNA profiles of EVs secreted during hatching in IVP and IVV bovine embryos.

- IVV 7–9 group: Blastocysts obtained via superovulation and artificial insemination.
- IVP 7–9 group: Blastocysts generated through in vitro fertilization.

In both experiments, the culture media from blastocysts on d 7 and d 9 were collected and stored at −80 °C for subsequent EV and content analysis.

EV characterization included size and concentration assessment via nanoparticle tracking analysis (NTA) and surface marker detection (CD9, CD40, CD63, and CD81) using cytometry and transmission electron microscopy (TEM). Additionally, miRNA profiles within the EVs were evaluated through sequencing. All blastocysts were transferred to a new drop of EV-depleted synthetic oviduct fluid (SOF) medium and maintained in individual culture until d 11 to assess their post-hatching developmental capacity. Only conditioned media from these competent embryos were used for the analysis.

The IETS classification system was applied at each stage of embryo development. Only embryos graded 1–2 on d 5 and 9 of development were considered for further study. Morphological evaluation was performed under blind conditions, in accordance with IETS guidelines. After classification, embryos were randomly and blindly assigned to the corresponding experimental groups. Subsequently, embryos that showed linear growth and a diameter greater than 270 µm on d 11 were considered competent [24].

### Bovine embryo in vitro production

Cumulus-oocyte complexes (COCs) were obtained from ovaries collected from slaughtered beef cattle at a local abattoir (Frigosur, Chillán, Chile). The collection, maturation, fertilization, and culture procedures followed the protocols described by [25, 31]. COCs were aspirated from antral follicles (3–6 mm diameter) using a 19-G needle. COCs were first transferred to manipulation medium (TCM199 supplemented with 4 mmol/L bicarbonate, 18 mmol/L HEPES, 10% fetal bovine serum (FBS), and 50 mg/mL gentamicin), pre-warmed to 38 °C, for washing and morphological assessment. Selected COCs were matured in groups in 500 μL of TCM199-based medium supplemented with 0.6 mmol/L glutamine, 0.2 mmol/L pyruvate, 0.01 IU/mL FSH and LH, 1 μg/mL estradiol, 50 μg/mL gentamicin, 10 ng/mL epidermal growth factor (EGF), and 10% FBS. Cultures were incubated at 38 °C in a humidified atmosphere of 5% CO_2_ for 20–22 h.

Spermatozoa were obtained from commercial semen (Semex, Madison, WI, USA) and separated using a Percoll gradient method. Each matured oocyte was inseminated with 10,000 motile spermatozoa in 500 μL of fertilization medium (TALP-FIV) supplemented with 0.01 mg/mL heparin, 2 mmol/L pyruvate, 50 mg/mL gentamicin, and 6 mg/mL fatty acid-free serum albumin (BSA). Fertilization was carried out at 39 °C under 5% CO_2_.

Eighteen hours after fertilization, presumptive zygotes were mechanically denuded of cumulus cells by vortexing for 3 min in TCM199 medium containing HEPES and 0.3 mg/mL hyaluronidase. Zygotes were then cultured in groups of 25–30 in 500 μL of SOF medium supplemented with 0.37 mmol/L trisodium citrate, 2.77 mmol/L myo-inositol, 10 ng/mL EGF, 2% FBS, and 3 mg/mL fatty acid-free BSA.

For Exp. 1, on d 5 of development, Grade 1 morula-stage embryos were selected and cultured individually in 96-well plates containing 80 µL of EV-depleted SOF culture medium. Incubation was maintained until d 7 of development, after which the embryos were evaluated and classified based on morphological quality, and the culture medium was collected. In Exp. 2, on d 7, the embryos were classified. Grade I blastocysts were then selected and cultured individually in 96-well plates with 80 µL of EV-depleted SOF culture medium until d 9 of development. On d 9, the embryos were re-evaluated, classified by morphological quality, and the culture medium was collected for further analysis. In both cases, the blastocysts were transferred to fresh SOF medium for continued monitoring until d 11 of development. Incubation was carried out at 38.5 °C under a gas mixture of 5% CO_2_, 5% O_2_, and 90% N_2_, with a humidity of 85%.

### Bovine in vivo embryo production

The donor females underwent assessment of follicular populations via ultrasonography (Sonoscape X3V with a 5 MHz sector transducer). To synchronize the emergence of the follicular wave among the donors, a 1.38-g intravaginal progesterone device (CIDR^®^; Zoetis) combined with 2.5 mg of estradiol benzoate (Zoetis) administered intramuscularly and 50 mg of progesterone administered intramuscularly [32, 33]. Hormonal stimulation for superovulation (SOV) began on d 4 and continued until d 7. Each donor in the control group received a total of 250 mg of NIH-FSH-P1 (Folltropin-V; Bioniche Animal Health, Belleville, Ontario, Canada), divided into eight intramuscular injections with decreasing doses from 50 to 10 mg, administered every 12 h over 4 d.

On d 6, two doses of prostaglandin F_2α_ (cloprostenol, Ciclase^®^, Zoetis), each 500 µg, were administered intramuscularly in the morning and evening, with an interval of 12 h. The CIDR devices were removed 12 h before the last NIH-FSH-P1 injection on d 7 [34]. Following the onset of estrus, artificial insemination was performed at 12 h and again at 48 h after estrus detection. Embryos were collected from donor females on d 5 and 7 after the onset of estrus using a closed transrectal uterine lavage system [35]. Uterine catheters of the Foley silicon type with two channels were used, along with commercial collection media ABT360 Complete Flush [36].

### Developmental monitoring and sample collection

Embryos were monitored daily from d 5 to d 11 post-fertilization. Developmental stage and embryo quality were assessed at each time point using the IETS classification system, and only grade 1–2 embryos were included in the analysis. Classification as a blastocyst or a hatched blastocyst was assigned based on cumulative developmental progression over the culture period rather than on a single endpoint assessment.

Conditioned media were collected from grade 1–2 embryos at d 5 and d 9 post-fertilization, as described in the experimental design. In both experiments, blastocysts were transferred to fresh SOF medium and maintained under the same incubation conditions until d 11 of development.

### EV isolation and characterization

#### Isolation

Culture media from each group were individually processed for EV purification using the protocol described by Théry et al. [37] and validated in our laboratory by Mellisho et al. [25]. The procedure involves sequential centrifugation designed to remove cells and debris. Initially, samples were centrifuged at 700 × *g* for 20 min to remove cellular debris. The supernatant was then subjected to ultracentrifugation at 120,000 × *g* for 120 min using a fixed-angle rotor (PAOST-2201) in an ultracentrifuge (CP 80 NX; Hitachi Koki Co., Ltd., Tokyo, Japan). The resulting pellet, containing EVs, was resuspended in 500 μL of phosphate-buffered saline (PBS). The isolated EVs were characterized according to the criteria established by the International Society for Extracellular Vesicles (ISEV).

#### Size and concentration analysis of particles

The characterization of EVs isolated from the culture media of IVP and IVV-derived bovine embryos was performed using NTA with the NanoSight NS300 system (Malvern Instruments Ltd., Malvern, UK). For the analysis, the nanoparticle pellet was resuspended in sterile, filtered PBS and diluted 1:20 in PBS. Each individual sample was loaded into 1-mL tuberculin syringes and connected to the NanoSight syringe pump to ensure a constant flow rate during measurements. Samples were analyzed in triplicate under consistent camera settings: an acquisition time of 60 s, a detection threshold set at level 8, and a 488 nm laser. Filtered sterile PBS was used as a negative control to rule out external contamination. In addition, EV-depleted SOF culture medium, prepared by ultracentrifugation under the same conditions, was analyzed without embryos as an additional negative control [38].

#### Analysis of molecular markers

Specific EV markers (CD9, CD63, CD81, and CD40L), as defined by the ISEV [19], were evaluated by flow cytometry, following the protocol described by Mellisho et al. [25]. Nanoparticles were incubated with 4 μm aldehyde/sulfate latex beads (1.25 × 10^5^ particles/mL; ThermoFisher Scientific, Waltham, MA, USA). The nanoparticle-bead complexes were incubated with primary antibodies against CD63 (FITC-conjugated; catalog No. 18235, Abcam), CD9 (FITC-conjugated; catalog No. 34162, Abcam), CD81 (PE-conjugated; catalog No. 81436, Abcam), and CD40L (PE/Cy5^®^-conjugated; catalog No. 25044, Abcam) for 2 h at 4 °C. Negative controls included latex beads incubated with antibodies alone, and EV-depleted SOF medium incubated with the same antibodies under identical conditions. Both controls were analyzed in parallel. The absence of signal confirmed antibody specificity and the embryonic origin of the detected EVs. Samples were analyzed using an Attune™ NxT Flow Cytometer (ThermoFisher Scientific).

#### Transmission electron microscopy (TEM)

EVs isolated from embryo culture media were prepared for TEM imaging using four copper grids, following the protocols described by Théry et al. [39]. Initially, 200-mesh copper grids coated with Formvar-carbon were floated on a 50 μL drop of isolated EV suspension for 3 min. The grids were then washed in three consecutive 50 μL drops of PBS to remove excess EVs and subsequently fixed in a 50 μL drop of 1% glutaraldehyde in 0.1 mol/L PBS. For negative staining, the grids were placed on a 50 μL drop of uranyl oxalate solution (Electron Microscopy Sciences, USA; pH 7.0) for 5 min, then transferred to a 50 μL drop of methylcellulose-uranyl acetate solution (Sigma M6385) for 10 min on an ice-cold surface. Grids were then air-dried overnight at room temperature. Finally, each grid was mounted on the sample holder of a TEM, and EVs were visualized at magnifications ranging from 40,000 × to 80,000 ×. Approximately five images per sample were captured and processed using ImageJ software (v1.47t, NIH, USA; https://imagej.nih.gov/ij/download.html).

### EV RNA isolation and quantification

For each experimental group, three biological replicates were prepared, each consisting of a pool of 10 conditioned culture media samples. The experimental groups included IVV 5–7, IVV 7–9, IVP 5–7, and IVP 7–9. Total RNA was extracted using the Exosomal RNA Isolation Kit (Norgen Biotek, Thorold, Canada) according to the manufacturer's instructions and following the protocols described by Lässer et al. [40]. RNA integrity was assessed with the RNA ScreenTape Assay (Agilent Technologies, Santa Clara, CA, USA). Next-generation sequencing (NGS) was outsourced to an external facility (Norgen Biotek, Thorold, Canada).

### Small RNA sequencing and data analysis

Small RNA libraries were prepared using the Small RNA Library Prep Kit (Norgen Biotek, Thorold, Canada), according to the manufacturer’s instructions. Sequencing was performed on an Illumina NextSeq 500 platform with the NextSeq 500/550 High Output Kit v2 (Illumina, San Diego, CA, USA). Library quality was evaluated using FastQC (Babraham Bioinformatics, Cambridge, UK). Adapter sequences were trimmed, reads aligned, and quantified using the SRNA bench pipeline [41]. For miRNA analysis, reads with a Phred score above 20 and lengths between 18 and 30 base pairs were accepted. Reads were mapped against the reference genome ARS-UCD 1.2 and miRBase version 21 using Bowtie2 and miRDeep2 mapper. Gene counts were calculated with HTSeq, applying a cutoff of counts per million (CPM) ≥ 5 to filter low-abundance features.

### Statistical and bioinformatic analyses

Embryonic morphological characteristics and measurable EV variables (mean size and concentration) for each experimental group were compared using the Mann–Whitney U test. The correlation between embryo quality scores and developmental competence was assessed via Pearson’s and Spearman’s correlation tests. Differential expression analysis of the miRNA profiles was performed using the EdgeR package. The analysis included normalization of read counts using the trimmed mean of M-values (TMM) method. Statistical significance was determined using the exact test implemented in EdgeR, and *P*-values were adjusted for multiple testing using the Benjamini–Hochberg false discovery rate (FDR) correction. MiRNAs were considered differentially expressed based on an adjusted FDR < 0.05 and an absolute log₂ fold change > 1.2 (or < −1.2). The results were presented as volcano plots, heat maps, and principal component analysis (PCA) plots.

The set of differentially expressed miRNAs was further analyzed for target gene prediction using miRinGO. Gene Ontology (GO) enrichment analysis was performed via the DAVID platform to identify biological processes and pathways associated with the miRNA targets. A venn diagram was constructed using Venny 2.1 (https://bioinfogp.cnb.csic.es/tools/venny).

## Results

### Embryo development

Bovine pre-implantation embryos produced IVV and IVP were analyzed at two developmental windows: blastulation (d 5–7) and hatching (d 7–9) (Table 1). Across both windows, IVV embryos exhibited significantly higher developmental competence than IVP embryos. During the blastulation window, a greater proportion of IVV embryos reached the blastocyst stage between d 5 and 7, as determined by daily individual tracking. This difference was maintained at the hatching window (d 7–9), with IVV embryos also showing a markedly higher hatching rate (*P* < 0.05). In contrast, developmental progression to d 11 was substantially reduced in IVP embryos compared with their IVV counterparts.

**Table 1:** Development and progression of bovine embryos produced in vitro (IVP) and in vivo (IVV) during the blastulation (d 5–7) and hatching (d 7–9) stages

Groups	Number of presumptive zygotes	Number of collected embryos	% morulae (n)	% blastocyst (n)	% hatched blastocyst (n)
IVP 5–7	612		44.9 (275)^a^	24.7 (151)^a^	10.5 (64)^a^
IVV 5–7		61	78.7 (48)^b^	63.9 (39)^b^	52.5 (32)^b^
IVP 7–9	701		42.2 (289)^a^	26.7 (187)^a^	13.1 (92)^a^
IVV 7–9		73	83.6 (61)^b^	71.2 (52)^b^	61.6 (45)^b^

^a,b^Different superscript letters within the same column indicate statistically significant differences between groups (*P* < 0.05). Percentages reflect the number of embryos that reached at each any point of evaluation during the culture period (d 5–11), based on individual daily tracking

**Fig. 2:**
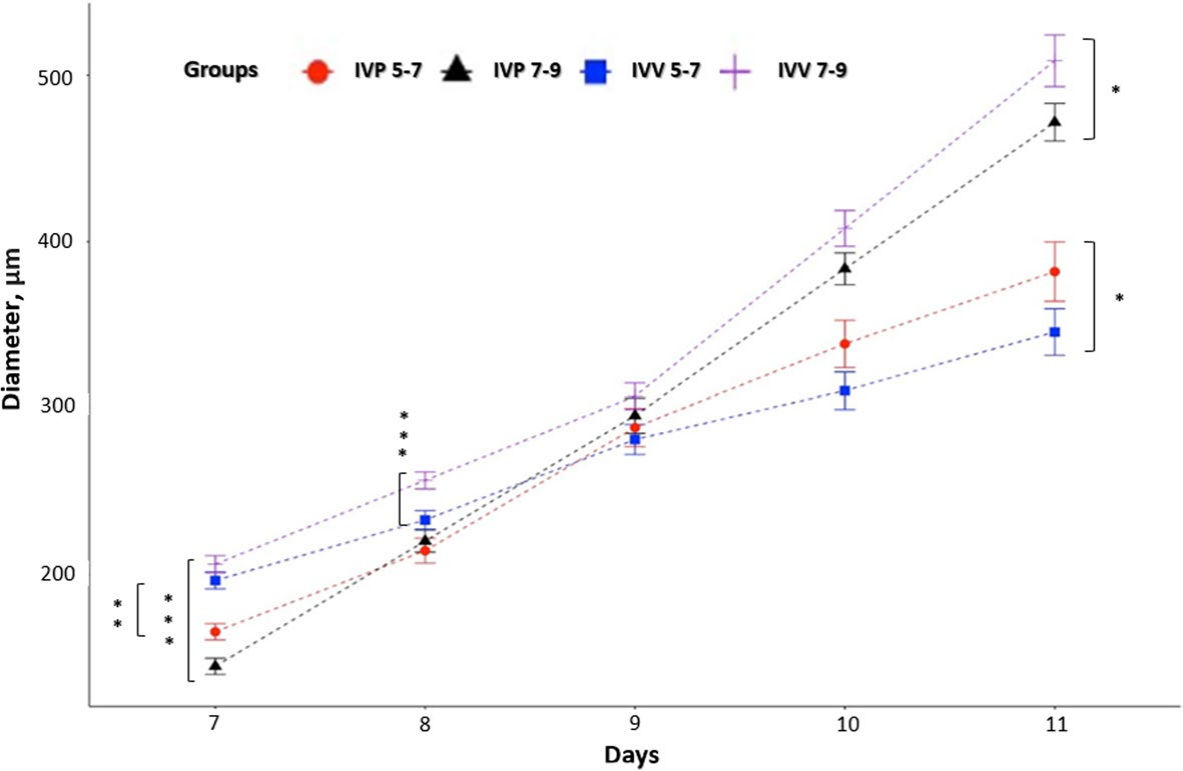
Kinetics of bovine embryo development from d 7 to d 11. Developmental progression of individually cultured bovine blastocysts produced in vitro (IVP) or in vivo (IVV) from d 7 to d 11 post-fertilization. Values represent the mean ± SEM of three independent replicates and based on individual daily tracking. Statistical differences between groups are indicated by ^*^P < 0.05, **P < 0.01, ***P < 0.001

Embryonic growth analysis revealed differences that were dependent on both developmental stage and embryonic origin (Fig. 2). Embryos at d 7–9 reached significantly larger diameters than those at d 5–7. Throughout the entire culture period, IVV embryos consistently exhibited greater growth than IVP embryos (*P* < 0.05). Embryo quality was assessed using the IETS classification system, and only embryos graded 1–2 were considered developmentally competent. Based on these, 133 conditioned media samples derived from competent embryos were selected for EVs analysis.

### EV characterization

**Fig. 3:**
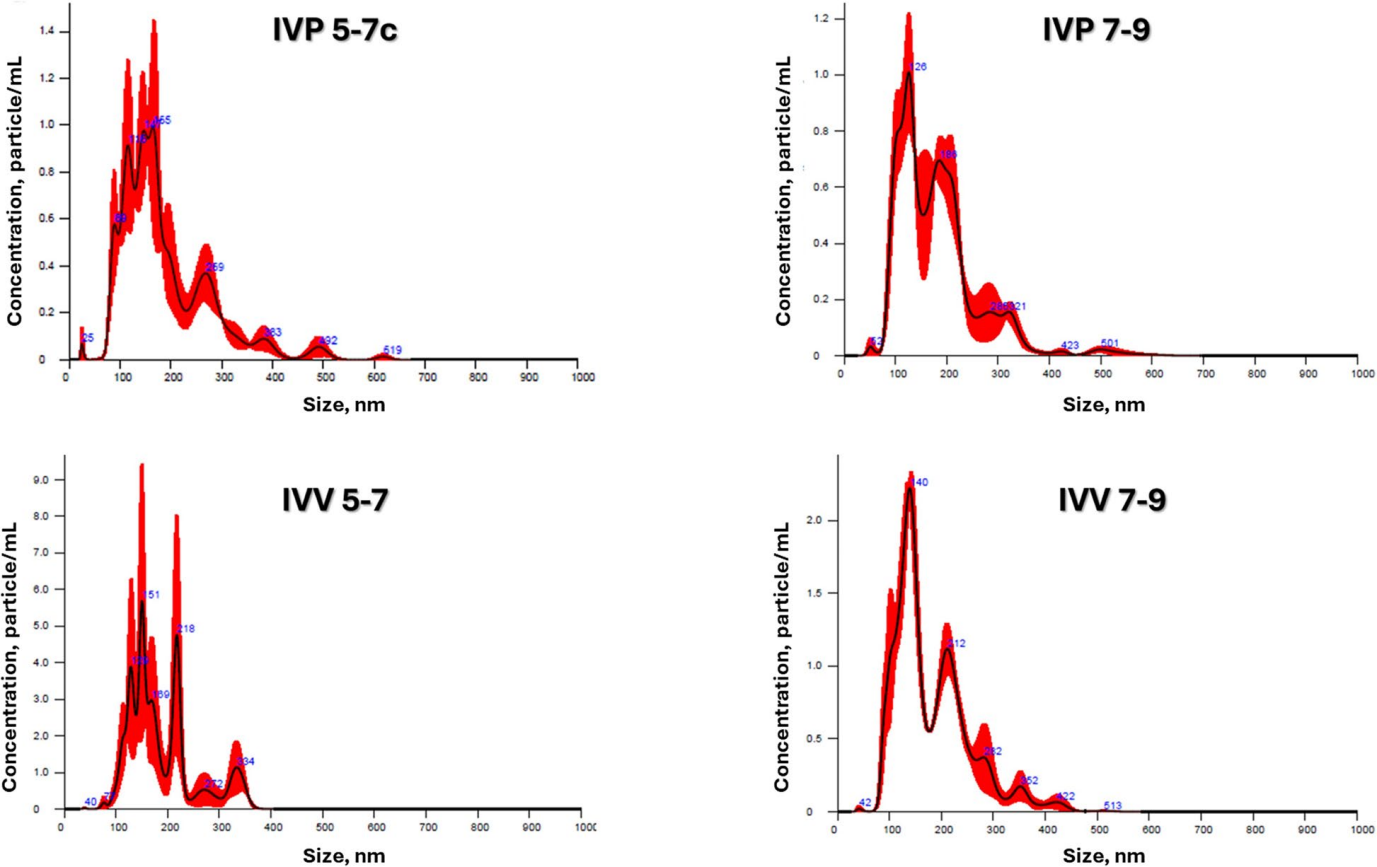
Extracellular vesicle (EV) concentration and size distribution determined by nanoparticle tracking analysis (NTA, NanoSight^©^). EVs were isolated from embryo-conditioned media collected from bovine embryos produced in vivo (IVV) or in vitro (IVP) during the blastulation (d 5–7) and hatching (d 7–9) stages. Each curve represents the average particle size (nm) and concentration (particles/mL) from three replicates per group. SOF medium was used as control

NTA analysis showed that EVs from all experimental groups corresponded to the size range expected for embryonic EVs (Fig. 3). EV concentration and size distribution were significantly influenced by embryonic origin and developmental stage (Table 2). IVP embryos secreted a higher number of EVs than IVV embryos, particularly during the hatching stage (IVP 7–9; *P* < 0.05). In contrast, IVV embryos displayed more stable EV concentrations across stages.

**Table 2:** Concentration and size parameters of extracellular vesicles (EVs) secreted by bovine embryos during the blastulation (d 5–7) and hatching (d 7–9) stages under in vitro (IVP) and in vivo (IVV) conditions

**EV parameters**	**Blastulation**		**Hatching**		
	**IVP 5–7**	**IVV 5–7**	**IVP 7–9**	**IVV 7–9**	**SOFdep**
Concentration, particles/mL	2.9 × 10^9^ ± 0.3^a(§)^	2.6 × 10^9^ ± 0.8^a^	3.8 × 10^9^ ± 0.3^b(ţ)^	2.2 × 10^9^ ± 0.3^a^	1.6 × 10^7^ ± 3.5 × 10^6c(ȵ)^
Size, nm	124.7 ± 3.1^a(§)^	148.5 ± 4.1^b(§)^	118.1 ± 2.1^a(ţ)^	115.4 ± 1.8^a(ţ)^	114.7 ± 6.6 ^a(ţ)^

^a–c^Superscript letters indicate statistically significant differences between embryo origins within the same stage (*P* < 0.05). Symbols (§, ţ, ȵ) indicate statistically significant differences between developmental stages within the same embryo origin (*P* < 0.05). EV-depleted SOF: EV-depleted culture medium, used as a negative control for NTA. The depleted medium contained 12 ± 0.2 particles/frame, whereas the samples contained more than 50 particles/frame

**Fig. 4:**
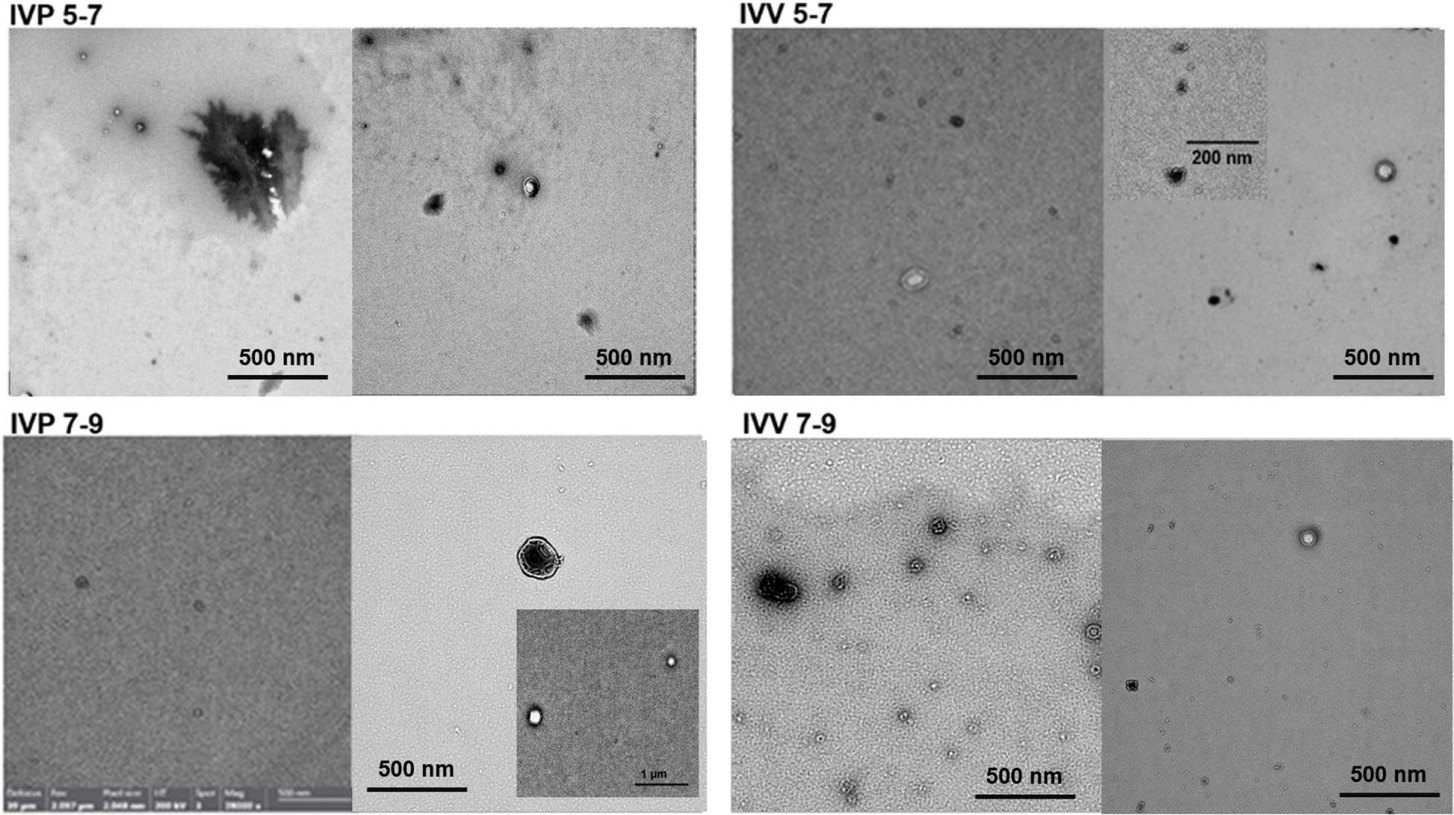
Morphology of extracellular vesicles (EVs) secreted by bovine embryos under different experimental conditions. Representative TEM images of EVs isolated from embryo-conditioned media of bovine embryos produced in vitro (IVP) or in vivo (IVV) during blastulation (d 5–7) and hatching (d 7–9) stages. Scale bars are included in each picture

At the blastulation stage, EVs derived from IVV embryos were significantly larger than those from IVP embryos (*P* < 0.05), whereas no size differences were detected during hatching. The presence of EV-specific surface markers (CD9, CD63, CD81, and CD40) was confirmed in all groups by flow cytometry (Table 3), and transmission electron microscopy revealed typical EV morphology without structural differences among groups (Fig. 4).

**Table 3:** Characterization of surface markers on extracellular vesicles (EVs) secreted by bovine embryos produced in vitro (IVP) and in vivo (IVV) during the blastulation and hatching stages

Groups	Surface markers (positive percentage), %			
	**CD9**	**CD63**	**CD81**	**CD40**
IVP 5–7	10.5	24.4	36.6	9.3
IVV 5–7	3.5	7.6	3.8	20.8
IVP 7–9	17.9	3.9	15.7	9.2
IVV 7–9	7.4	13.0	7.8	18.5
Bovine blood serum (positive control)	14.5	6.5	9.6	12.0
EV-depleted SOF (negative control)	0.7	0.2	0.5	0.9
Beads with antibodies (negative control)	0	0	0	0

Values represent the percentage of positivity for surface markers CD9, CD63, CD81, and CD40 on EVs, as determined by flow cytometry

### miRNA cargo analysis

**Fig. 5:**
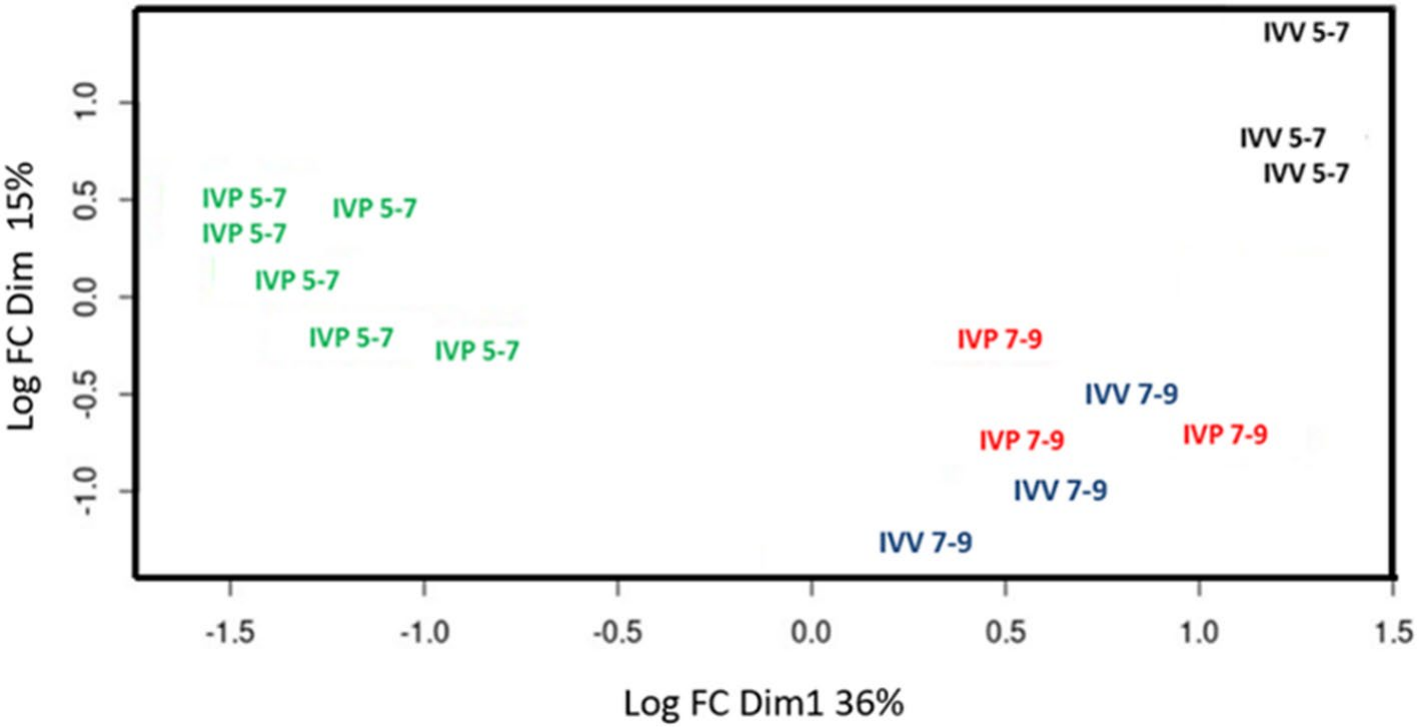
Principal component analysis (PCA) of miRNA profiles in extracellular vesicles (EVs) secreted by bovine embryos. The plot represents the dimensional distribution of EV-associated miRNA expression profiles according to embryo origin and developmental stage. Colors denote experimental groups: Green, IVP 5–7; Black, IVV 5–7; Red, IVP 7–9; and Blue, IVV 7–9. Axes represent the principal components calculated from log_2_ fold-change (Log FC) values. Each point corresponds to an independent experimental replicate

Principal component analysis demonstrated a clear separation of EV-associated miRNA profiles according to embryonic origin, with IVV and IVP-derived EVs forming distinct clusters across both developmental stages (Fig. 5). Embryonic origin accounted for most of the variance, while developmental stage contributed to secondary dispersion.

**Fig. 6:**
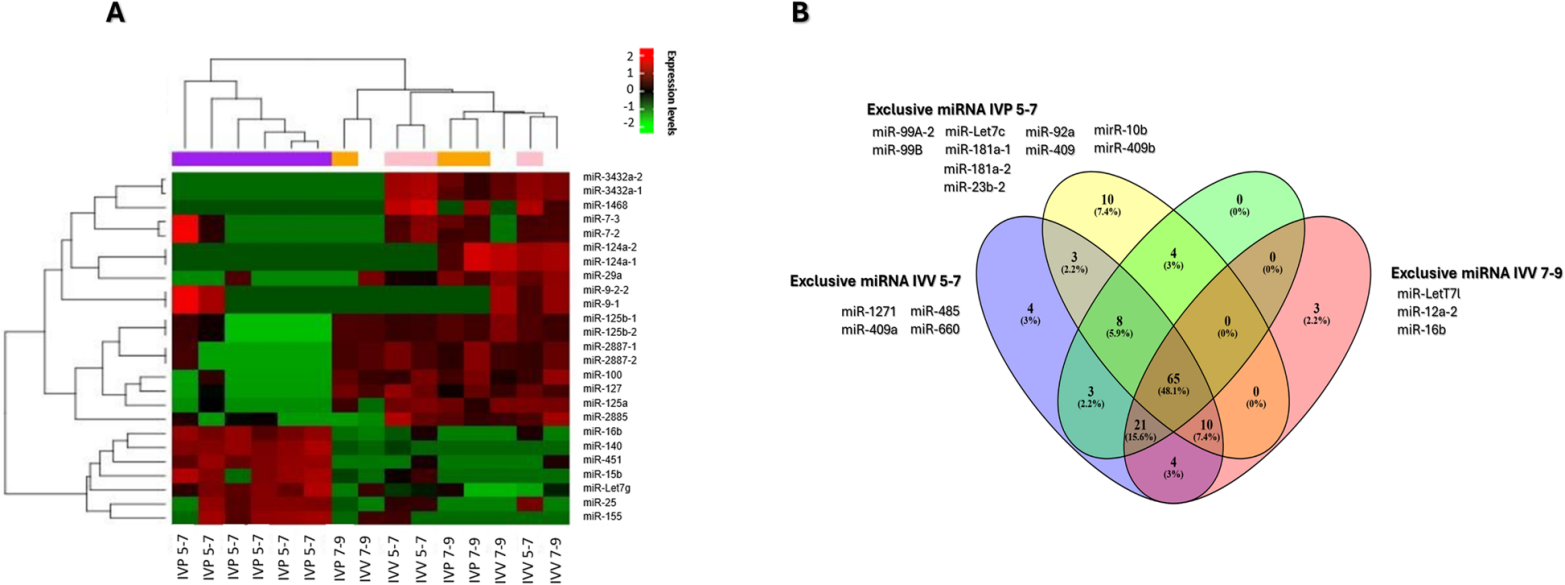
Distribution and expression profiles of miRNAs in extracellular vesicles (EVs) from bovine embryos produced in vitro (IVP) and in vivo (IVV) during blastulation (5–7) and hatching (7–9). **A** Heatmap representing the hierarchical clustering of miRNA expression levels identified in EVs secreted by bovine embryos. Each row corresponds to a specific miRNA, and each column to an experimental replicate (IVP 5–7, IVV 5–7, IVP 7–9, IVV 7–9). The color scale indicates relative expression levels; Red: upregulated miRNAs, Green: downregulated miRNAs. Dendrograms depict clustering based on the similarity of expression profiles. **B** Venn diagram showing the distribution and overlap of miRNAs detected in EVs according to embryonic origin and developmental stage. Numbers within each area represent the number and percentage of miRNAs shared or uniquely expressed among the groups IVP 5–7, IVV 5–7, IVP 7–9, IVV 7–9

A total of 122 miRNAs were identified in EVs, of which 65 were shared among all groups, constituting a conserved miRNA core (Fig. 6B). Distinct miRNA signatures were associated with specific origins and stages, with IVP embryos, particularly at the blastulation stage, displaying the most divergent profiles (Fig. 6A).

### Prediction of gene functions

**Fig. 7:**
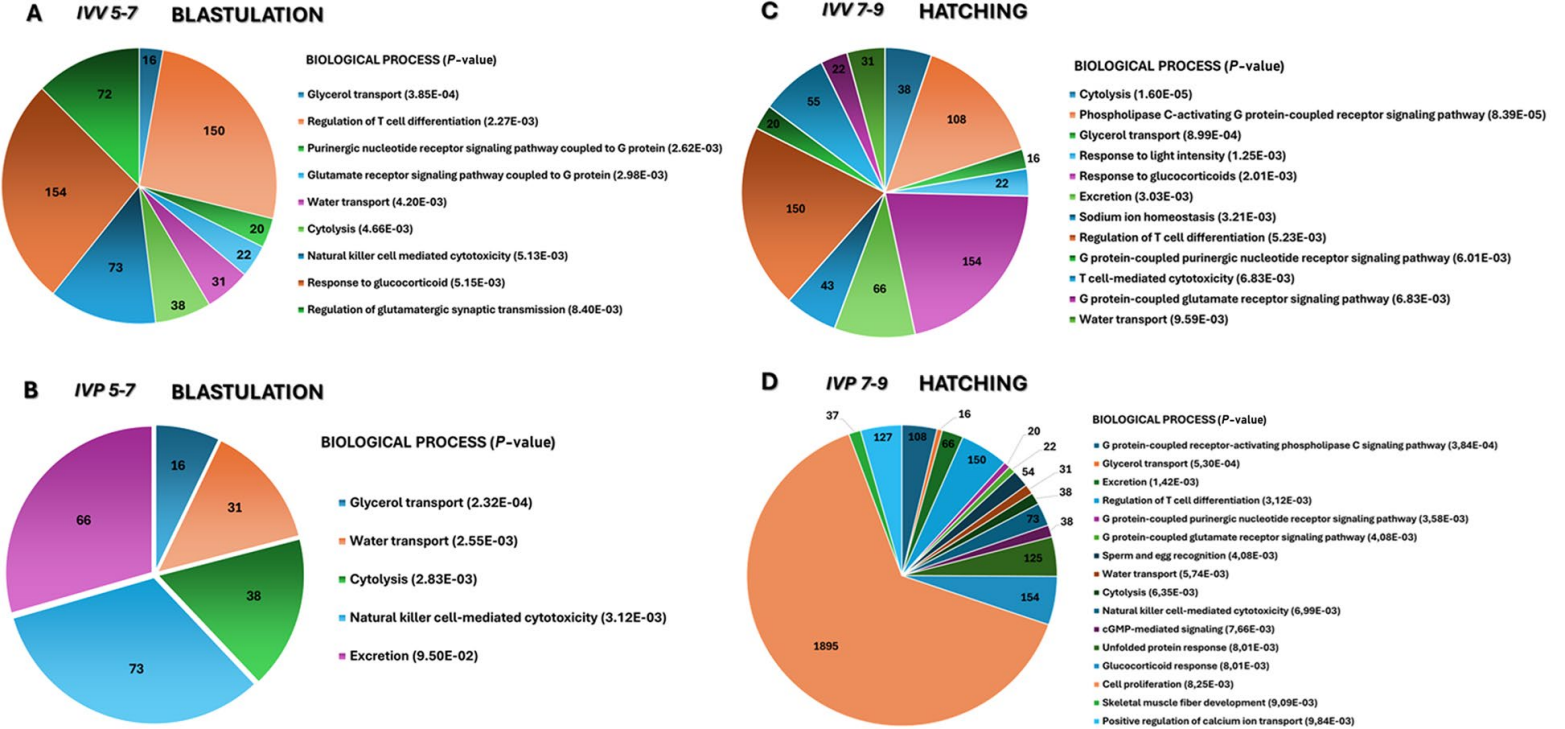
Biological processes regulated by differentially expressed miRNAs in extracellular vesicles (EVs) from bovine embryos during blastulation and hatching under in vivo (IVV) and in vitro (IVP) conditions. Pie charts illustrate the distribution of genes associated with significantly enriched biological processes (Gene Ontology analysis). The number within each segment indicates the count of genes involved in each process, while *P*-values (shown in parentheses). **A** IVV 5–7 (blastulation). **B** IVP 5–7 (blastulation). **C** IVV 7–9 (hatching). **D** IVP 7–9 (hatching)

Gene Ontology analysis revealed that EV-associated miRNAs regulate biological processes that differ according to embryonic origin and developmental stage (Fig. 7A–D). During blastulation, both IVV and IVP embryos showed enrichment in water and glycerol transport pathways, while IVV embryos uniquely exhibited immune-related and signaling processes. At the hatching stage, IVV embryos maintained enrichment in homeostatic and cytolytic functions, whereas IVP embryos showed increased representation of pathways associated with stress responses, calcium regulation, and cell proliferation.

**Fig. 8:**
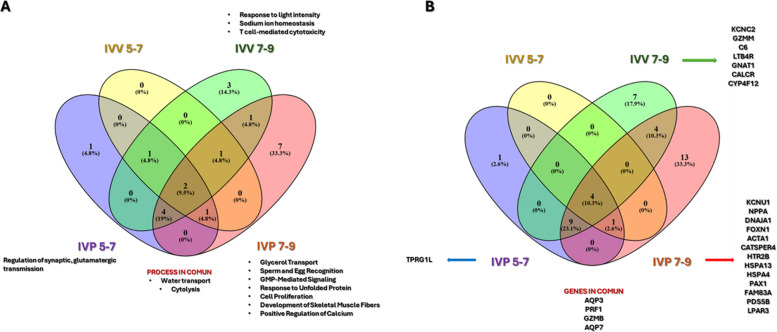
Distribution and interaction of biological processes and genes regulated by overexpressed miRNAs in extracellular vesicles (EVs) secreted during bovine embryonic development under in vivo (IVV) and in vitro (IVP) conditions. **A** Venn diagram showing the distribution of biological processes regulated by overexpressed miRNAs according to embryo origin and developmental stage. Shared and exclusive biological processes are indicated, with the number and percentage of processes represented within each region. **B** A Venn diagram illustrates the distribution of genes targeted by overexpressed miRNAs. Shared and exclusive genes are shown for each condition. Specific biological processes and genes associated with each experimental group are listed adjacent to the diagrams: Yellow, IVV 5–7; Blue, IVP 5–7; Green, IVV 7–9; and Red, IVP 7–9

Gene-level analysis indicated progressive diversification of predicted EV-associated targets toward the hatching stage, particularly in IVP embryos (Fig. 8B). Four genes (*AQP3*,* PRF1*, *GZMB*, and *AQP7*) were shared across all groups, indicating a conserved core of EV-associated signaling.

## Discussion

This study provides a comprehensive and comparative characterization of EVs secreted by bovine embryos produced IVV and IVP during two critical stages of preimplantation development: blastulation and hatching. Previous studies have shown that IVP embryos secrete EVs with distinctive characteristics and potential value as biomarkers of developmental viability [18, 25, 42]. Other research has indicated that embryonic origin can also modulate the miRNA profile of EVs in hatched blastocysts examined at a single developmental stage [43]. In this context, our work substantially expands previous knowledge by demonstrating that the population characteristics and miRNA repertoire of EVs depend simultaneously on embryo origin and developmental stage, reflecting differential molecular programs that may influence early embryo–maternal communication.

IVV embryos showed higher developmental competence across both developmental windows, as evidenced by increased blastocyst formation, higher hatching rates, and sustained post-hatching survival. These observations are consistent with previous studies reporting altered developmental kinetics, reduced robustness, and increased sensitivity to environmental stress in IVP embryos [3, 44]. In line with this interpretation, embryo manipulation and culture-associated stress have been shown to influence both developmental progression and EV secretion dynamics, supporting the notion that EV release may function as a sensitive readout of embryo physiological state and adaptive responses to environmental change [24, 43].

Once the differential competence of embryos from different groups was confirmed, the impact on the released EV population was assessed. EV size decreased as embryonic development progressed, regardless of origin, although this reduction was more pronounced in IVV embryos. This pattern contrasts with reports in porcine and human embryos, where EV size increases during preimplantation development [25, 45, 46]. The largest EVs were detected in IVV embryos collected at d 5, which subsequently showed reduced growth, suggesting that early embryo recovery may impose stress affecting both developmental dynamics and EV populations. While Mellisho et al. [25] reported an inverse association between EV size and embryo quality; their analyses were limited to IVP embryos and single developmental windows.

EV concentration remained relatively stable in IVV embryos across blastulation and hatching, whereas IVP embryos showed a progressive increase in EV release. Embryos produced IVP exhibited higher EV concentrations, particularly at later stages, supporting the view that the type of culture promotes EV secretion as part of a stress-adaptation response [43]. Unlike previous single-window studies, the inclusion of both developmental stages reveals that the effect of embryonic origin on EV concentration is stage-dependent, and that EV size dynamics diverge across systems when interpreted alongside embryo competence and growth kinetics [47, 48].

The analysis of miRNA cargo within embryonic EVs has gained relevance, as these molecules exert post-transcriptional control over essential developmental processes, including pluripotency, cellular differentiation, and tissue remodeling [49–51]. Multiple studies have shown that the miRNA cargo of EVs varies with embryo origin and developmental competence [17, 24, 52]. This supports a functional role for EVs in embryo–maternal communication, allowing the preimplantation embryo to signal its developmental state and contribute to endometrial receptivity.

PCA revealed a clear separation of EV miRNA profiles according to embryonic origin, consistent with previous observations that IVV and IVP-derived embryos exhibit distinct molecular signatures. Embryos of different origins have been shown to secrete EVs with distinct miRNA profiles during the d 7–9 window, with these variations linked to pathways involved in embryo–maternal recognition [29, 43, 53]. Our results extend these findings by demonstrating that, beyond embryonic origin, developmental stage acts as an additional and independent axis of variability. This stage-dependent remodeling of EV miRNA cargo suggests a coordinated regulation of molecular signals that may modulate embryo–maternal communication through dynamic adaptation of secreted vesicular content.

Moreover, the higher within-group dispersion observed in IVP embryos supports the notion that in vitro culture imposes heterogeneous molecular states, consistent with earlier observations of increased transcriptomic and epigenetic variability under artificial culture conditions [42, 54, 55]. These heterogeneous molecular signatures may be selectively packaged into EVs, as proposed for embryo-derived and reproductive tract EVs involved in embryo–maternal signaling [17, 43, 56].

Global miRNA profiling identified a subset of highly variable miRNAs associated with pathways essential for implantation and early embryonic development. Several of these miRNAs, including miR-1468-5p, miR-7, miR-124, and miR-29a, have been consistently detected in embryonic EVs and are known to regulate cell proliferation, apoptosis, and differentiation [57–59]. Mechanistically, these miRNAs modulate key developmental pathways, such as TGF-β/Smad signaling, lineage specification, and stromal differentiation, providing a molecular basis for their involvement in early embryo development and uterine preparation [60–63].

Additional EV-associated miRNAs further reinforce the multifunctional nature of embryonic signaling. MiR-9 and miR-127 regulate cell adhesion, vasculogenesis, and mesendoderm differentiation through E-cadherin/VEGF and Nodal/Smad2 pathways, respectively [64, 65]. MiR-155 contributes to osmotic regulation via AQP3/AQP7 and to immune tolerance through Treg modulation [66]. Notably, the exclusive detection of miR-let-7g in in vivo–derived embryos supports its role in coordinating embryonic growth with uterine receptivity through repression of c-Myc/mTOR signaling, a mechanism previously linked to implantation success [67, 68].

Together, these miRNAs cluster into three main functional axes: regulation of pluripotency and differentiation, embryonic competence, and immune modulation related to embryonic viability. The identification of a conserved core of miRNAs across all experimental groups suggests the existence of a foundational EV-mediated signaling program that is maintained across embryonic origins and developmental stages. In parallel, origin and stage-specific miRNAs indicate adaptive modulation of EV cargo in response to developmental context and environmental conditions [17, 69].

During blastulation (d 5–7), EV miRNA profiles were dominated by conserved processes related to transport and osmoregulation, including glycerol and water transport, which are essential for blastocoel formation. However, within this conserved framework, IVP embryos exhibited unique miRNAs such as let-7c, miR-181a, miR-23b, and miR-409, which are associated with cell proliferation, oxidative stress responses, Wnt/β-catenin and MAPK/p38 signaling [70–74]. The exclusive detection of miR-409-3p in IVP embryos suggests activation of compensatory developmental pathways not observed in IVV embryos, consistent with adaptation to in vitro culture conditions [74]. At the functional level, IVP embryos during blastulation displayed exclusive enrichment of glutamatergic synaptic transmission pathways. Although uncommon in embryonic contexts, NMDA receptor–related signaling has been implicated in cellular differentiation in other developmental systems [75, 76]. Its detection in IVP embryos may therefore reflect alternative regulatory mechanisms engaged under culture-associated stress.

As embryos progressed toward hatching (d 7–9), EV miRNA profiles suffered further remodeling. IVP embryos did not exhibit exclusive miRNAs during this window, suggesting partial convergence of EV cargo as development advances, as previously reported [60]. Nevertheless, IVP embryos showed the highest degree of functional diversification, with enrichment of pathways related to glycerol transport, GMP-mediated signaling, calcium regulation, and the unfolded protein response. The overexpression of molecular chaperones (DNAJA1, HSPA13, HSPA4) [77, 78] and regulators of Ca^2+^ signaling (e.g., CATSPER4) [79, 80] indicates activation of stress-adaptive mechanisms aimed at maintaining cellular homeostasis and blastocoel integrity under suboptimal conditions [81].

In contrast, EVs derived from IVV embryos during hatching exhibited a more constrained functional profile, characterized by enrichment of sodium homeostasis and immune-related pathways. The expression of KCNC2, CALCR, GZMM, and C6 highlights the importance of ionic regulation for sustained blastocoel expansion and suggests early immunomodulatory signaling rather than cytotoxic activity [82, 83]. These processes are consistent with coordinated embryo–maternal communication during the peri-implantation period and may contribute to the superior implantation potential of IVV embryos.

Across all groups, shared biological processes converged on water transport and signaling related to cytolysis. The consistent identification of AQP3 and AQP7 supports their central role in blastocoel expansion, particularly during the transition to hatching [84, 85]. The presence of cytolysis-associated genes such as *PRF1* and *GZMB* [86, 87] in EV cargo likely reflects immunomodulatory priming of the maternal environment rather than effector cytotoxicity, in line with previous reports of embryo-derived EV–mediated regulation of endometrial immune responses [88].

Overall, these findings demonstrate that embryonic origin modulates EV-mediated embryo–maternal communication in a stage-dependent manner. EVs are dynamically regulated according to developmental context, reflecting a balance between adaptive signaling in IVP embryos and implantation-oriented communication in IVV embryos. By extending previous studies limited to single developmental stages or exclusively in vitro-derived embryos [24, 26, 43]. This work supports the view that embryonic EVs function as active signaling entities that integrate developmental stage and origin to shape early molecular dialogue with the maternal endometrium.

## Conclusions

This study confirms that both the origin (IVV vs. IVP) and developmental stage (blastulation vs. hatching) of bovine embryos critically influence the characteristics and molecular composition of EVs, particularly their miRNA cargo. IVV embryos exhibit superior developmental competence, more stable EV profiles, and miRNA signatures linked to ionic balance and controlled immunomodulation. In contrast, IVP embryos exhibit increased EV release, greater heterogeneity in miRNA profiles, and activation of stress-related pathways, suggesting adaptive mechanisms in response to suboptimal culture conditions.

The observed divergence in EV cargo and associated biological processes reveals that EVs play a dynamic role in pre-implantation embryo–maternal communication. These differences have profound implications for reproductive biotechnology, as they provide a potential non-invasive biomarker platform to assess embryo quality and competence. Future research should focus on functionally validating the predicted targets of EV-associated miRNAs and on evaluating their impact on maternal endometrial responses. Such insights will contribute to the development of novel diagnostic and therapeutic strategies to enhance implantation success and pregnancy rates in assisted reproduction programs.

## Supplementary Information

Additional file 1. miRNA expression profiles detected in extracellular vesicles (EVs) secreted by bovine embryos produced in vivo (IVV) and in vitro (IVP) during the blastulation (d 5–7) and hatching (d 7–9) stages.

## Data Availability

The datasets generated and analyzed during the current study, including miRNA profiles and EV characterization data, are available from the corresponding author upon reasonable request.

## Abbreviations

Ca^2+^: Calcium ion
CPM: Counts per million
EMT: Epithelial–mesenchymal transition
EVs: Extracellular vesicles
GO: Gene Ontology
GMP: Cyclic guanosine monophosphate
IVP: In vitro-produced
IVV: In vivo-derived
Log FC: Log_2_ fold change
miRNA/miRNAs: MicroRNA(s)
NTA: Nanoparticle tracking analysis
PCA: Principal component analysis
SEM: Standard error of the mean
TEM: Transmission electron microscopy
TGF-β: Transforming growth factor beta
UPR: Unfolded protein response
