# Supplementary material for: Comparative profiling of extracellular vesicles and miRNA cargo from in vivo- and in vitro-derived bovine embryos during blastulation and hatching
Source: J Anim Sci Biotechnol. 2026 Apr 13;17:66. doi: 10.1186/s40104-026-01378-y (PMC13072587; doi:10.1186/s40104-026-01378-y)
Supplement: Supplementary file 1 — Additional file 1. miRNA expression profiles detected in extracellular vesicles (EVs) secreted by bovine embryos produced in vivo (IVV) and in vitro (IVP) during the blastulation (d 5–7) and hatching (d 7–9) stages. [file 40104_2026_1378_MOESM1_ESM.docx]

**Additional file 1**. **miRNA expression profiles detected in extracellular vesicles (EVs) secreted by bovine embryos produced in vivo (IVV) and in vitro (IVP) during the blastulation (Days 5–7) and hatching (Days 7–9) stages.**

| **miARNs** | **CPM**  **IVP 5-7** |  | **miARNs** | **CPM**  **IVV 5- 7** |  | **miARNs** | **CPM**  **IVP 7-9** |  | **miARNs** | **CPM**  **IVV 7- 9** |
| --- | --- | --- | --- | --- | --- | --- | --- | --- | --- | --- |
| miR-9-1 | 1968,65 |  | miR-6529a | 2538,68 |  | miR-30d | 1174,30 |  | miR-6529a | 1922,67 |
| miR-9-2-2 | 1968,65 |  | miR-6529b | 2538,68 |  | miR-10b | 13447,69 |  | miR-6529b | 1922,67 |
| miR-30d | 4401,44 |  | miR-9-1 | 259,57 |  | miR-409b | 1996,63 |  | miR-9-1 | 2327,82 |
| mirR-10b | 25218,18 |  | miR-9-2-2 | 259,57 |  | miR-92b | 2507,46 |  | miR-9-2-2 | 2327,82 |
| mirR-409b | 140,44 |  | miR-30d | 5846,01 |  | miR-29a | 3512,18 |  | miR-30d | 7721,30 |
| miR-92a | 231,30 |  | miR-10b | 97826,75 |  | miR-92a-1 | 8952,96 |  | miR-10b | 38380,69 |
| miR-29a | 280,88 |  | miR-409b | 4105,17 |  | miR-92a-2 | 8952,96 |  | miR-92b | 528,85 |
| miR-92a-1 | 18898,84 |  | miR-92b | 6569,22 |  | miR-215 | 4529,99 |  | miR-29a | 8373,98 |
| miR-92a-2 | 15827,14 |  | miR-29a | 2244,08 |  | miR-122 | 58661,40 |  | miR-92a-1 | 2347,27 |
| miR-122 | 1762,57 |  | miR-92a-1 | 16385,23 |  | miR-Let7f-1 | 5911,42 |  | miR-92a-2 | 2347,27 |
| miR-29b-2 | 421,32 |  | miR-92a-2 | 16366,10 |  | miR-184 | 35820,08 |  | miR-215 | 2634,36 |
| miR-29c | 421,32 |  | miR-215 | 181,68 |  | miR-423 | 23724,37 |  | miR-122 | 111415,38 |
| miR-29d-2 | 421,32 |  | miR-122 | 295003,83 |  | miR-148a | 10890,18 |  | miR-29b-2 | 1110,53 |
| miR-29e | 421,32 |  | miR-Let7f-1 | 4873,89 |  | miR-186 | 25,21 |  | miR-29c | 1110,53 |
| miR-Let7f-1 | 20731,62 |  | miR-184 | 15279,06 |  | miR-Let7f-2 | 6817,09 |  | miR-29d-2 | 1110,53 |
| miR-184 | 13073,98 |  | miR-423 | 69170,35 |  | miR-30c | 439,27 |  | miR-29e | 1110,53 |
| miR-423 | 2680,69 |  | miR-142-2 | 47,81 |  | miR-423-2 | 1491,67 |  | miR-Let7f-1 | 12657,31 |
| miR-142-2 | 1038,01 |  | miR-148a | 11886,25 |  | miR-Let7d | 1019,43 |  | miR-184 | 8268,49 |
| miR-148a | 5192,38 |  | miR-186 | 5297,91 |  | miR-375 | 629,17 |  | miR-423 | 37636,46 |
| miR-186 | 874,07 |  | miR-361 | 2885,80 |  | miR-658 | 5532,08 |  | miR-142-2 | 1079,37 |
| miR-361 | 125,35 |  | miR-Let7f-2 | 5292,22 |  | miR-103-1 | 1244,10 |  | miR-148a | 5162,37 |
| miR-Let7f-2 | 20850,69 |  | miR-30c | 105,18 |  | miR-103-2 | 1244,10 |  | miR-186 | 1083,66 |
| miR-30c | 482,54 |  | miR-423-2 | 2007,69 |  | miR-191 | 6799,04 |  | miR-361 | 918,98 |
| miR-423-2 | 867,60 |  | miR-Let7d | 62,19 |  | miR-Let7g | 1296,02 |  | miR-Let7f-2 | 12668,28 |
| miR-Let7d | 1951,24 |  | miR-375 | 284,31 |  | miR-23a | 108,42 |  | miR-30c | 4,33 |
| miR-375 | 406,23 |  | miR-658 | 2185,74 |  | miR-183 | 14813,70 |  | miR-423-2 | 2955,05 |
| miR-103-1 | 1050,33 |  | miR-103-1 | 513,95 |  | miR-200b | 1323,01 |  | miR-Let7d | 10,97 |
| miR-103-2 | 1050,33 |  | miR-103-2 | 513,95 |  | miR-107 | 840,92 |  | miR-375 | 26,27 |
| miR-191 | 34423,07 |  | miR-191 | 23172,98 |  | miR-128-1 | 747,91 |  | miR-658 | 1064,24 |
| miR-451 | 14001,29 |  | miR-451 | 688,73 |  | miR-128-2 | 747,91 |  | miR-191 | 9630,50 |
| miR-Let7g | 8308,50 |  | miR-Let7g | 184,02 |  | miR-143 | 9267,81 |  | miR-451 | 702,24 |
| miR-148b | 119,06 |  | miR-148b | 414,20 |  | miR-144 | 507,58 |  | miR-Let7g | 3135,83 |
| miR-23a | 1947,33 |  | miR-23a | 1752,61 |  | miR-205 | 503,32 |  | miR-148b | 1085,79 |
| miR-183 | 12798,86 |  | miR-183 | 10582,54 |  | miR-2285k-1 | 428,58 |  | miR-23a | 1699,23 |
| miR-185 | 2593,14 |  | miR-185 | 449,41 |  | miR-2285k-4 | 428,58 |  | miR-183 | 8057,61 |
| miR-128-1 | 231,30 |  | miR-200b | 760,59 |  | miR-320a-1 | 22675,24 |  | miR-185 | 438,70 |
| miR-128-2 | 370,71 |  | miR-107 | 513,95 |  | miR-320a-2 | 22675,24 |  | miR-200b | 1288,49 |
| miR-143 | 4068,16 |  | miR-128-1 | 1659,95 |  | miR-146b | 7705,64 |  | miR-143 | 5566,72 |
| miR-144 | 1327,46 |  | miR-128-2 | 792,99 |  | miR-222 | 469,70 |  | miR-205 | 10,97 |
| miR-205 | 1365,43 |  | miR-143 | 5858,99 |  | miR-26a-1 | 10903,81 |  | miR-320a-1 | 31134,43 |
| miR-320a-1 | 7674,47 |  | miR-144 | 9,56 |  | miR-26a-2 | 10903,81 |  | miR-320a-2 | 31134,43 |
| miR-320a-2 | 7674,47 |  | miR-205 | 473,21 |  | miR-26c | 10903,81 |  | miR-146b | 2640,60 |
| miR-146b | 2926,26 |  | miR-2285k-1 | 2413,40 |  | miR-455 | 781,53 |  | miR-222 | 4,33 |
| miR-222 | 469,42 |  | miR-2285k-4 | 2413,40 |  | miR-30b | 16,81 |  | miR-26a-1 | 7781,33 |
| miR-26a-1 | 10074,76 |  | miR-320a-1 | 9656,63 |  | miR-21 | 3890,68 |  | miR-26a-2 | 7781,33 |
| miR-26a-2 | 10074,76 |  | miR-320a-2 | 9656,63 |  | miR-2889 | 6888,88 |  | miR-26c | 7781,33 |
| miR-26c | 10074,76 |  | miR-146b | 2459,20 |  | miR-100 | 36968,18 |  | miR-30b | 2516,99 |
| miR-455 | 1105,93 |  | miR-222 | 23,94 |  | miR-151 | 7785,39 |  | miR-21 | 4049,34 |
| miR-30b | 1231,07 |  | miR-26a-1 | 20189,84 |  | miR-30a | 369,97 |  | miR-2889 | 23297,83 |
| miR-21 | 6132,37 |  | miR-26a-2 | 20189,84 |  | miR-10a | 23468,10 |  | miR-100 | 42589,83 |
| miR-2889 | 708,23 |  | miR-26c | 20189,84 |  | miR-30e | 5199,38 |  | miR-151 | 7112,65 |
| miR-100 | 4054,52 |  | miR-455 | 357,79 |  | miR-378-2 | 4494,08 |  | miR-30a | 1506,37 |
| miR-151 | 3244,21 |  | miR-30b | 28,69 |  | miR-101-1 | 41,19 |  | miR-10a | 12733,14 |
| miR-30a | 3878,91 |  | miR-21 | 3161,05 |  | miR-101-2 | 41,19 |  | miR-30e | 2400,69 |
| miR-10a | 24256,95 |  | miR-2889 | 50495,76 |  | miR-1246 | 6682,39 |  | miR-378-2 | 2056,97 |
| miR-30e | 1813,56 |  | miR-100 | 34842,06 |  | miR-3596 | 12642,03 |  | miR-101-1 | 1062,03 |
| miR-378-2 | 1653,18 |  | miR-151 | 19161,54 |  | miR-Let7b | 12642,03 |  | miR-101-2 | 1062,03 |
| miR-101-1 | 482,54 |  | miR-30a | 1677,10 |  | miR-LetT7l | 928,40 |  | miR-1246 | 7080,56 |
| miR-101-2 | 482,54 |  | miR-10a | 20321,72 |  | miR-1-1 | 959,45 |  | miR-3596 | 12781,88 |
| miR-1246 | 3126,87 |  | miR-30e | 796,11 |  | miR-1-2 | 959,45 |  | miR-Let7b | 12781,88 |
| miR-3596 | 12407,34 |  | miR-378-2 | 6216,16 |  | miR-7-3 | 1511,84 |  | miR-Let7I | 2181,20 |
| miR-Let7b | 12407,34 |  | miR-101-1 | 1939,71 |  | miR-124a-1 | 4174,57 |  | miR-1-1 | 45,30 |
| miR-Let7I | 11463,51 |  | miR-101-2 | 1939,71 |  | miR-12a-2 | 4174,57 |  | miR-1-2 | 45,30 |
| miR-1-1 | 1219,18 |  | miR-1246 | 5467,73 |  | miR-124b | 4174,57 |  | miR-7-3 | 515,84 |
| miR-1-2 | 1219,18 |  | miR-3596 | 10486,10 |  | miR-125a | 4390,74 |  | miR-124a-1 | 3121,17 |
| miR-7-3 | 2865,71 |  | miR-Let7b | 10486,10 |  | miR-125b-1 | 12407,95 |  | miR-124a-2 | 3121,17 |
| miR-125a | 119,06 |  | miR-Let7I | 727,55 |  | miR-125b-2 | 12407,95 |  | miR-124b | 3121,17 |
| miR-125b-1 | 1162,92 |  | miR-1-1 | 721,69 |  | miR-15b | 25,21 |  | miR-125a | 6175,84 |
| miR-125b-2 | 1162,92 |  | miR-1-2 | 721,69 |  | miR-182 | 12812,96 |  | miR-125b-1 | 17616,01 |
| miR-151-2 | 1351,37 |  | miR-7-3 | 3174,26 |  | miR-192 | 6368,46 |  | miR-125b-2 | 17616,01 |
| miR-155 | 119,06 |  | miR-124a-1 | 1045,30 |  | miR-22 | 325211,43 |  | miR-182 | 10147,61 |
| miR-15b | 2795,29 |  | miR-124a-2 | 1045,30 |  | miR-2285k-2 | 428,58 |  | miR-192 | 5930,63 |
| miR-182 | 14262,16 |  | miR-124b | 1045,30 |  | miR-2285k-3 | 428,58 |  | miR-22 | 230454,14 |
| miR-192 | 1512,02 |  | miR-125a | 11225,84 |  | miR-2285k-5 | 428,58 |  | miR-2285k-2 | 572,20 |
| miR-22 | 33636,11 |  | miR-125b-1 | 10566,73 |  | miR-24-2 | 1413,12 |  | miR-2285k-3 | 572,20 |
| miR-24-2 | 1315,81 |  | miR-125b-2 | 10547,61 |  | miR-2478 | 665,45 |  | miR-2285k-5 | 572,20 |
| miR-25 | 3454,65 |  | miR-1271 | 2570,24 |  | miR-25 | 25,21 |  | miR-24-2 | 1088,19 |
| miR-26b | 1211,87 |  | miR-151-2 | 1897,94 |  | miR-26b | 25,21 |  | miR-2478 | 322,06 |
| miR-27a-2 | 7504,16 |  | miR-155 | 749,61 |  | miR-27a-2 | 11784,54 |  | miR-27a-2 | 9871,97 |
| miR-27b | 10651,48 |  | miR-15b | 648,86 |  | miR-27b | 17074,78 |  | miR-27b | 13823,54 |
| miR-2887-1 | 343,33 |  | miR-182 | 9050,23 |  | miR-2887-1 | 8266,14 |  | miR-2887-1 | 5528,20 |
| miR-2887-2 | 343,33 |  | miR-192 | 18055,80 |  | miR-2887-2 | 8266,14 |  | miR-2887-2 | 5528,20 |
| miR-3600 | 33636,11 |  | miR-22 | 121796,94 |  | miR-2898 | 1692,21 |  | miR-2898 | 490,60 |
| miR-378-1 | 279,85 |  | miR-2285k-2 | 2793,63 |  | miR-3432a-1 | 1104,34 |  | miR-3432a-1 | 2052,65 |
| miR-409 | 377,54 |  | miR-2285k-3 | 2793,63 |  | miR-3432a-2 | 1422,53 |  | miR-3432a-2 | 2052,65 |
| miR-486 | 158578,08 |  | miR-2285k-5 | 2793,63 |  | miR-3600 | 325211,43 |  | miR-3600 | 230454,14 |
| miR-7-2 | 2865,71 |  | miR-24-2 | 1613,57 |  | miR-378-1 | 2416,21 |  | miR-378-1 | 2453,03 |
| miR-99A-2 | 2278,46 |  | miR-2478 | 223,82 |  | miR-486 | 7511,45 |  | miR-486 | 7654,18 |
| miR-99B | 4644,61 |  | miR-25 | 2920,85 |  | miR-7-2 | 1026,99 |  | miR-7-2 | 515,84 |
| miR-127 | 119,06 |  | miR-26b | 19,12 |  | miR-99a-2 | 20338,24 |  | miR-99a-2 | 22017,00 |
| miR-140 | 3383,48 |  | miR-27a-2 | 3851,21 |  | miR-99b | 2210,44 |  | miR-99b | 10757,91 |
| miR-16B | 5260,13 |  | miR-27b | 11922,71 |  | miR-127 | 6093,77 |  | miR-127 | 3497,53 |
| miR-2890 | 357,19 |  | miR-2887-1 | 7020,40 |  | miR-140 | 8,40 |  | miR-1468 | 255,75 |
| miR-Let7a-1 | 14725,16 |  | miR-2887-2 | 7020,40 |  | miR-1468 | 1519,30 |  | miR-2890 | 6467,25 |
| miR-Let7a-2 | 14725,16 |  | miR-2898 | 296,42 |  | miR-16b | 8,40 |  | miR-Let7a-1 | 6042,14 |
| miR-Let7a-3 | 14725,16 |  | miR-3432a-1 | 9448,28 |  | miR-2890 | 7289,12 |  | miR-Let7a-2 | 6042,14 |
| MIR-Let7c | 3867,34 |  | miR-3432a-2 | 9448,28 |  | miR-Let7a-1 | 9698,51 |  | miR-Let7a-3 | 6042,14 |
| MIR-181a-1 | 2341,50 |  | miR-3600 | 121796,94 |  | miR-Let7a-2 | 9698,51 |  | miR-Let7c | 4513,80 |
| MIR-181a-2 | 2341,50 |  | miR-378-1 | 3338,98 |  | miR-Let7a-3 | 9698,51 |  | miR-181a-1 | 2408,02 |
| MIR-23b-2 | 1170,59 |  | miR-409a | 301,66 |  | miR-Let7c | 5711,24 |  | miR-181a-2 | 2408,02 |
|  |  |  | miR-485 | 1135,84 |  | miR-181a-1 | 1997,71 |  | miR-23b-2 | 416,77 |
|  |  |  | miR-486 | 8779,70 |  | miR-181a-2 | 1997,71 |  |  |  |
|  |  |  | miR-660 | 1320,18 |  | miR-23b-2 | 129,62 |  |  |  |
|  |  |  | miR-7-2 | 3179,07 |  |  |  |  |  |  |
|  |  |  | miR-99a-2 | 16410,12 |  |  |  |  |  |  |
|  |  |  | miR-99b | 11062,97 |  |  |  |  |  |  |
|  |  |  | miR-127 | 11407,45 |  |  |  |  |  |  |
|  |  |  | miR-140 | 28,69 |  |  |  |  |  |  |
|  |  |  | miR-1468 | 9366,07 |  |  |  |  |  |  |
|  |  |  | miR-16B | 896,16 |  |  |  |  |  |  |
|  |  |  | miR-2890 | 5986,79 |  |  |  |  |  |  |
|  |  |  | miR-Let7a-1 | 7109,45 |  |  |  |  |  |  |
|  |  |  | miR-Let7a-2 | 7119,01 |  |  |  |  |  |  |
|  |  |  | miR-Let7a-3 | 7358,06 |  |  |  |  |  |  |
|  |  |  | miR-Let7c | 3666,15 |  |  |  |  |  |  |
|  |  |  | miR-181a-1 | 2819,06 |  |  |  |  |  |  |
|  |  |  | miR-181a-2 | 2819,06 |  |  |  |  |  |  |
|  |  |  | miR-23b-2 | 47,81 |  |  |  |  |  |  |

**CPM:** normalized readings in log₂ counts per million
